# Beyond Mortality: Textbook Outcome as a Novel Quality Metric in Cardiothoracic Surgical Care

**DOI:** 10.3390/jcm14217660

**Published:** 2025-10-28

**Authors:** Dimitrios E. Magouliotis, Vasiliki Androutsopoulou, Prokopis-Andreas Zotos, Ugo Cioffi, Fabrizio Minervini, Noah Sicouri, Dimitrios Zacharoulis, Andrew Xanthopoulos, Marco Scarci

**Affiliations:** 1 Department of Cardiac Surgery Research, Lankenau Institute for Medical Research, Wynnewood, PA 19096, USA; dimitrios.magouliotis.18@alumni.ucl.ac.uk; 2 Department of Cardiothoracic Surgery, University of Thessaly, Biopolis, 41110 Larissa, Greece; androutsopoulouvasiliki@uth.gr (V.A.); zotospro@hotmail.com (P.-A.Z.); 3 Department of Surgery, University of Milan, 20157 Milan, Italy; ugo.cioffi@guest.unimi.it; 4 Department of Thoracic Surgery, Luzern Kanton Hospital, 6000 Luzern, Switzerland; fabrizio.minervini@luks.ch; 5 Department of Neuroscience Pittsburgh Campus, University of Pittsburgh, Pittsburgh, PA 15260, USA; nps67@pitt.edu; 6 Department of Surgery, University of Thessaly, Biopolis, 41110 Larissa, Greece; zacharoulis@uth.gr; 7 Department of Cardiology, University of Thessaly, Biopolis, 41110 Larissa, Greece; andrewvxanth@gmail.com; 8 Department of Cardiothoracic Surgery, Hammersmith Hospital, Imperial College Healthcare, National Health Service (NHS) Trust, London W2 1NY, UK

**Keywords:** textbook outcome, thoracic surgery, quality metrics, lung cancer, esophagectomy, transplantation

## Abstract

**Objective:** Textbook outcome (TO) is a multidimensional composite quality metric that integrates several desirable short-term outcomes into a single measure representing the “ideal” perioperative course. Unlike traditional indicators that focus narrowly on mortality or single complications, TO captures the complexity of cardiothoracic care, encompassing oncologic adequacy, absence of major complications, avoidance of reintervention and readmission, and timely discharge. **Methods:** In this systematic review, we synthesize evidence on the definition, incidence, determinants, prognostic impact, and limitations of TO across cardiothoracic surgery (lung and esophageal resections, lung transplantation, cardiac surgery, and adult heart transplantation) in accordance with the PRISMA guidelines. **Results:** Reported achievement rates range from 24% to 66% in thoracic series, 30% after Norwood palliation, 37–45% after adult heart transplantation, and 52% in a contemporary national cohort of lung transplantation, with wide between-center variability. Achieving TO is consistently associated with improved overall and disease-free survival, lower costs, and enhanced benchmarking. Determinants of failure include inadequate lymph node dissection, prolonged operative time, advanced comorbidity, pretransplant organ support, and socioeconomic disadvantage. Heterogeneity of definitions, limited incorporation of patient-reported outcomes, and equity concerns remain barriers to its successful use. Outside transplantation, benchmarking of TO in adult cardiac procedures (e.g., CABG/valve) remains limited and non-standardized. **Conclusions:** We argue for harmonized, procedure-specific core TO sets aligned with widely available registry fields, integration of equity-sensitive risk adjustment, and prospective validation. TO is poised to become a cornerstone metric of quality assessment and improvement in cardiothoracic surgery.

## 1. Introduction

Quality assessment in cardiothoracic surgery has long rested on narrow endpoints such as operative mortality, specific complication rates, or length of stay. These measures, while essential, can be blunt in modern practice where mortality is low and morbidity is multifactorial. Over the last decade, failure to rescue (FTR) has been proposed as a promising composite quality metric [1,2]. Yet FTR is a reactive signal, dependent on a complication occurring, and vulnerable to coding and case mix effects, whereas textbook outcome (TO) proactively captures prevention of harm, oncologic adequacy, and timely recovery in a single patient-centered composite. The concept of TO emerged in gastrointestinal surgery to address these limitations by defining a composite endpoint that represents an “ideal” postoperative course with regard to multiple complementary dimensions [3]. Methodologically, TO is attractive because it aggregates clinically meaningful events into a single, intuitive indicator whose binary nature simplifies interpretation and benchmarking [4]. Whereas FTR emphasizes a program’s ability to mitigate harm once a major complication has occurred, TO is proactive, rewarding episodes that avoid complications, achieve oncologic adequacy, and support timely recovery in a single composite. Conceptually, FTR is a reactive safety signal; TO is a patient-centered success signal. Both are informative, but TO may offer greater validity for benchmarking when mortality is rare and morbidity multifactorial, while FTR remains valuable for rescue-process audits.

In thoracic surgery, TO has been applied to non-small cell lung cancer (NSCLC) resections and esophagectomy, with early work demonstrating feasibility and prognostic significance [5,6,7]. Composite definitions typically include complete resection, adequacy of lymph node dissection, absence of major complications and early mortality, no prolonged hospitalization, and no early readmission. The concept has extended to lung transplantation, where transplant-specific elements, such as early graft function, ventilatory milestones, and freedom from grade-3 primary graft dysfunction (PGD3), can be captured in registry fields and combined to define a pragmatic, risk-adjustable TO [8,9]. National audits and institutional registries have also demonstrated the utility of TO in benchmarking outcomes across hospitals [10]. However, TO definitions remain heterogeneous and are not yet standardized across procedures and settings [11]. The present review synthesizes the current evidence on TO as a cardiothoracic quality metric. We summarize definitions and methodological variations; report incidence and reasons for failure; identify determinants and case mix factors; evaluate prognostic impact on overall and disease-free survival and graft outcomes; and discuss health-economic implications and between-hospital benchmarking. We also highlight limitations, particularly definitional heterogeneity and equity, before proposing directions for standardization and implementation.

## 2. Materials and Methods

### 2.1. Search, Article Selection, and Data Extraction Strategy

The present review followed a prospectively defined protocol aligned with Preferred Reporting Items for Systematic Reviews and Meta-Analyses (PRISMA) [12]. This systematic review was registered in the Open Science Framework (OSF) registry. The protocol is publicly available at the following DOI: https://doi.org/10.17605/OSF.IO/ESA9M; 25 September 2025. Our objective was to synthesize contemporary evidence on TO in cardiothoracic surgery, spanning lung and esophageal resections, lung transplantation, congenital heart surgery, and adult heart transplantation. A comprehensive search strategy was developed with a medical librarian. We interrogated PubMed/MEDLINE, Scopus, and the Cochrane Central Register of Controlled Trials from 1 January 2010 through 30 August 2025, reflecting the period of contemporary adoption of TO. To minimize retrieval bias, we screened ClinicalTrials.gov and the WHO ICTRP, scanned abstracts from major cardiothoracic societies, and performed forward citation chasing using Google Scholar. Search terms combined keywords and controlled vocabulary relating to “textbook outcome,” “composite outcome,” “quality metrics,” “thoracic surgery,” “lung cancer,” “esophagectomy,” “lung transplantation,” “congenital heart surgery,” and “heart transplantation.” Full database search strings and language filters will be provided in the Supplementary Materials.

Eligibility criteria were prespecified. We included original human studies evaluating TO (or closely related composites) in cardiothoracic surgery with explicit definitions and reporting on incidence, determinants, survival or graft outcomes, resource use, or benchmarking. Eligible designs comprised randomized trials, prospective and retrospective cohorts, registry analyses, and propensity-matched studies. We excluded case reports, small series (<10 patients), editorials, narrative reviews, methodological notes without primary data, non-cardiothoracic procedures, and reports lacking outcome data linked to the TO construct. Inclusion was restricted to English-language publications. In cases of overlapping cohorts, the most comprehensive or most recent report was selected.

Two reviewers (DEM, VA) independently screened titles/abstracts and assessed full texts, resolving disagreements by discussion with senior arbitration as needed. Inter-reviewer agreement was monitored during calibration. Data extraction, performed independently and in duplicate using a piloted form, captured study characteristics; setting and design; sample size; patient and procedural details; operational TO definition (and, where applicable, textbook oncological outcome); TO incidence and reasons for failure; determinants and risk-adjustment methods; perioperative and long-term outcomes (overall and disease-free survival, graft outcomes); resource use (length of stay, readmissions, costs/charges); and between-hospital variation. For continuous variables reported as medians with interquartile ranges, validated methods for mean/SD conversion were planned where quantitative synthesis was appropriate. Corresponding authors were to be contacted for clarifications or missing data when needed.

### 2.2. Quality and Publication Bias Assessment

**Figure 1:**
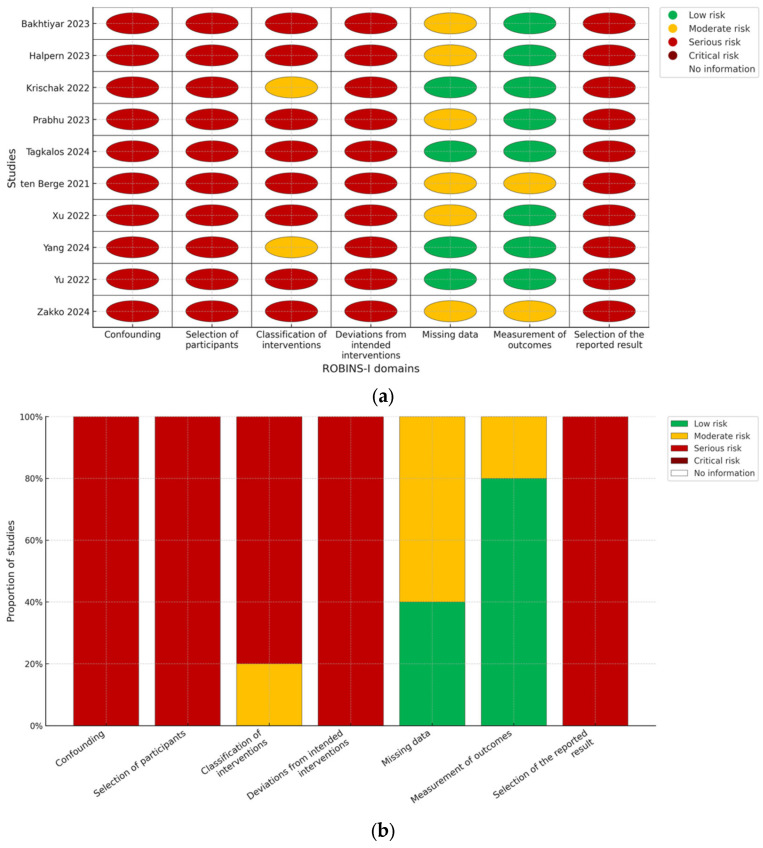
(**a**) Risk of bias summary (ROBINS-I). Domain-level judgments for each included study across the seven ROBINS-I domains: confounding; selection of participants; classification of interventions; deviations from intended interventions; missing data; measurement of outcomes; and selection of the reported result. Green = low risk; yellow = moderate; red = serious; white = no information. (**b**) Risk of bias overview (ROBINS-I). Proportion of studies at each risk level for every ROBINS-I domain. Green = low risk; yellow = moderate; red = serious; white = no information [5,6,7,8,14,15,16,17,18,19].

Risk of bias was assessed at the study level by two independent reviewers. No randomized trials were identified in the present review. For nonrandomized comparative studies and registry analyses, we used the ROBINS-I framework to appraise bias due to confounding, selection of participants, classification of interventions, deviations from intended interventions, missing data, outcome measurement, and selection of the reported result, assigning domain-level and overall judgments (low, moderate, serious, or critical) [13] (Figure 1). Certainty of evidence across key questions would be summarized narratively, with consideration of risk of bias, consistency, directness, precision, and reporting bias.

This study did not involve direct patient contact or access to identifiable information and therefore did not require institutional review board approval. All analyses were conducted using published, deidentified data from peer-reviewed articles and registries. Regarding risk of bias and reporting considerations, because all included studies were observational, we used ROBINS-I to appraise bias due to confounding, selection of participants, classification of interventions, deviations from intended interventions, missing data, measurement of outcomes, and selection of the reported result. Domain-level judgments (low, moderate, serious, or critical) were assigned in duplicate. In several studies, we judged a serious risk of “selection of the reported result” (selective reporting) when protocols were unavailable or when only a subset of predefined outcomes was presented. “Other bias” was used sparingly for concerns not captured by core domains (e.g., center-level learning-curve effects not adjusted in the analysis). We prespecified that these limitations would inform the certainty of evidence and would be explicitly acknowledged in the Discussion and reflected by conditional language in any practice implications.

## 3. Results

### 3.1. Definition of Textbook Outcome

**Figure 2:**
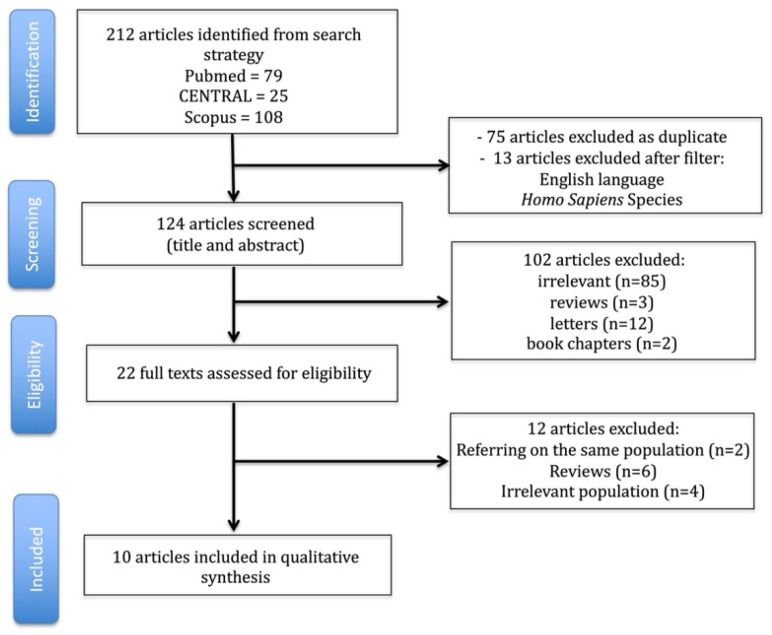
Flowchart of study selection for the current review.

A flowchart illustrating the literature search is presented in Figure 2 (Supplementary File S1). Definitions varied by procedure and data source (Table 1). In NSCLC resection, TO commonly encompasses complete (R0) resection; adequate lymph node dissection; absence of in-hospital/30-day mortality; no major complications or reintervention; no intensive care unit (ICU) readmission; no prolonged length of stay (often >14 days); and no 30-day readmission [5]. In esophagectomy, composite elements typically add oncologic thoroughness such as the retrieval of ≥15 lymph nodes, alongside perioperative endpoints (no ICU readmission, length of stay ≤21 days, no major complications, no early readmission) [6,7]. A large single-center minimally invasive esophagectomy (MIE) cohort emphasized the effect of operative time on TO and highlighted the sensitivity of TO to workflow and team factors [14].

In lung transplantation, single-center TO definitions included early extubation (≤48 h), freedom from PGD3 at 72 h, no ECMO or dialysis, and freedom from early rejection, reintubation, or tracheostomy, reflecting graft function and ventilatory milestones measurable in institutional datasets [8,9]. A contemporary national United Network for Organ Sharing (UNOS) analysis formalized a registry-based lung-transplant TO using standardized fields (e.g., intubated at 72 h, ECMO at 72 h, ventilator ≥5 days, PGD3 at 72 h, inpatient dialysis, airway dehiscence, 90-day mortality, index length of stay >30 days, 30-day readmission, pre-discharge acute rejection), enabling risk-adjusted between-center comparison [15].

In congenital cardiac surgery, an operation-specific TO for the Norwood procedure was defined via interdisciplinary consensus aligned with Society of Thoracic Surgeons (STS) elements: survival without ECMO, cardiac arrest, reintubation, reintervention, or 30-day readmission; invasive ventilation <10 days; and index length of stay <66 days [16]. In adult heart transplantation, two large Organ Procurement and Transplantation Network/United Network for Organ Sharing (OPTN/UNOS) analyses proposed complementary registry-based composites. One used a 10-item definition combining index hospitalization events (no stroke, dialysis, or treated rejection; length of stay ≤30 days) with 1-year patient-centered outcomes (left ventricular ejection fraction >50%, Karnofsky 80–100%, no 1-year rejection/graft failure/chronic dialysis/retransplantation/death) [17]. A second proposed a 6-domain framework emphasizing stricter index metrics (no ECMO within 72 h; length of stay <21 days) and 1-year endpoints (no readmission for rejection or infection; ejection fraction (EF) >50%), offering higher program-level discriminatory power than 1-year survival alone [18].

A related construct, textbook oncological outcome (TOO), integrates perioperative TO with oncologic process measures such as timely adjuvant therapy, thereby bridging surgical quality and comprehensive cancer care [20]. TOO, a construct integrating perioperative TO with oncologic process measures such as timely adjuvant therapy, was the primary focus in one included study (locally advanced NSCLC) and was referenced as a secondary construct in several thoracic series that otherwise reported TO components without a formal TOO composite. Where both appeared, TOO achievement tracked with improved survival but was less consistently reported than TO, underscoring the need for standard definitions and registry mapping.

**Table 1:** Representative definitions of textbook outcome in cardiothoracic surgery.

Procedure	Core Components of TO	Procedure-Specific Additions/Notes
NSCLC resection	R0 resection; adequate lymph node dissection; no in-hospital/30-day mortality; no major complications or reintervention; no ICU readmission; no prolonged length of stay (often >14 d); no 30-day readmission	Nodal adequacy was frequently the dominant driver of non-TO in population datasets [5,6]
Esophagectomy (MIE/open)	R0 resection; ≥15 nodes; no major complications; no ICU readmission; length of stay ≤21 d; no 30-day readmission	Radical lymphadenectomy often specified; TO sensitive to operative-time efficiency [7,10,14,19,21]
Lung transplantation (single-center)	Early extubation (≤48 h); no PGD3 at 72 h; no ECMO/dialysis; no early rejection; no reintubation/tracheostomy within 7 d; no major in-hospital complications	Transplant-specific ventilatory/graft milestones measurable in institutional datasets [8,9]
Lung transplantation (US registry)	Freedom from: intubation at 72 h; ECMO at 72 h; ventilation ≥5 d; PGD3 at 72 h; inpatient dialysis; airway dehiscence; 90-day mortality; index length of stay >30 d; 30-day readmission; pre-discharge acute rejection	Standardized UNOS fields enable national benchmarking and adjusted O:E TO rates [15]
Norwood operation (congenital)	Survival without ECMO, cardiac arrest, reintubation, or reintervention; no 30-day readmission; invasive ventilation <10 d; index length of stay <66 d	Operation-specific, consensus-derived composite aligned with STS elements [16]
Heart transplantation (adult, OPTN/UNOS 10-item)	Index: LOS ≤30 d; no stroke/dialysis/treated rejection. One-year: EF >50%, Karnofsky 80–100%, no treated rejection/graft failure/chronic dialysis/retransplant/death	Enables O:E TO rates for center benchmarking; TO associated with long-term survival [17]
Heart transplantation (adult, OPTN/UNOS 6-domain)	No ECMO ≤72 h; LOS <21 d; no postoperative stroke/pacemaker/dialysis; no PGD; no 1 y readmission for rejection/infection/re-transplant; EF >50% at 1 y	Higher discriminatory power than 1 y survival; quantifies inter-hospital variation [18]

Abbreviations: NSCLC, non-small cell lung cancer; MIE, minimally invasive esophagectomy; ICU, intensive care unit; LOS, length of stay; PGD, primary graft dysfunction; PGD3, primary graft dysfunction grade 3; ECMO, extracorporeal membrane oxygenation; UNOS, United Network for Organ Sharing; STS, Society of Thoracic Surgeons; OPTN/UNOS, Organ Procurement and Transplantation Network/United Network for Organ Sharing; EF, ejection fraction; O:E, observed-to-expected; re-TX, retransplantation.

### 3.2. Incidence of TO and Drivers of Failure

The incidence of TO across the included studies is demonstrated in Table 2. In NSCLC, the Dutch Lung Cancer Audit reported 26.4% of 5513 resections meeting TO, with inadequate lymph node dissection being the most common reason for failure [5]. In contrast, a Korean single-center study of 418 lobectomies reported a 66.3% TO rate, where prolonged air leak and prolonged length of stay were frequent drivers of non-TO [6].

In esophagectomy, TO achievement ranged from 40% to 53% depending on definition and cohort. A 528-patient MIE series for squamous cell carcinoma reported 53.2% TO with significantly improved five-year overall and disease-free survival among TO achievers [7]. A two-center European–Asian cohort reported 46.6%, with higher TO after robotic-assisted esophagectomy compared with thoracoscopy [19]. In a high-volume Chinese center (*n* = 2210), 40.8% achieved TO; operative time emerged as a critical and potentially modifiable driver of failure, showing an inverse-*U* relationship with TO likelihood [14].

In lung transplantation, an academic single-center series of 401 transplants found 24.2% achieved TO, most often limited by delayed extubation beyond 48 h [8]. In bilateral transplants without pulmonary hypertension, planned venoarterial ECMO support was associated with higher TO rates compared to off-pump strategies [9]. In contrast to these single-center estimates, a national UNOS analysis of 8959 adult lung transplants (2016–2019) reported a TO rate of 52.1%, with wide adjusted center-level variability (27.0–72.4%) [15]. The most frequent causes of non-TO in the UNOS dataset were intubation at 72 h (64.0%), index length of stay >30 days (50.5%), mechanical ventilation ≥5 days (44.1%), and PGD3 at 72 h (37.1%) [15]. The higher aggregate rate likely reflects definitional and ascertainment differences inherent to registry-specified fields and case mix differences in pretransplant support and procedure type compared with institutional series [8,9,15].

In cardiac surgery, the Norwood single-center cohort (*n* = 196) reported 30% TO; failure most commonly stemmed from prolonged ventilation (49%), reintubation (46%), and prolonged length of stay (30%) [16]. In adult heart transplantation, in a 24,620-patient OPTN/UNOS cohort (2005–2017), it was reported that 45.4% met a 10-item TO, while in a complementary 26,885-patient cohort (2011–2022), using a 6-domain construct, it was found that 37% met TO [17,18].

### 3.3. Determinants of TO and Risk Adjustment

Determinants can be grouped into patient-level factors, intraoperative/technical factors, and system-level factors. In esophagectomy, older age, higher American Society of Anesthesiologists (ASA) class, smoking, impaired pulmonary function, and greater blood loss reduced the likelihood of TO [6,7]. Operative time showed an inverse-U relationship with TO in MIE, peaking at approximately 298 min before declining, implicating team efficiency, ergonomics, and case complexity as potential levers for improvement [14]. In NSCLC, adequacy of lymph node dissection consistently determined TO, particularly in population datasets where nodal under-sampling was common [5]. Robotic-assisted approaches in esophagectomy were associated with higher TO rates than thoracoscopy, plausibly via enhanced visualization and ergonomics with fewer conversions [19].

In lung transplantation, patient and donor case mix was prominent. In the national UNOS analysis, pretransplant ventilator support and ECMO were associated with lower odds of TO; donation after circulatory death, longer ischemic time, obesity, and non-White race were likewise adverse predictors, whereas single-lung transplantation had higher odds than bilateral procedures [15]. In adult heart transplantation, not being hospitalized pretransplant and absence of preoperative mechanical support or dialysis predicted higher odds of TO [17,18]. In Norwood, greater weight increased odds of TO, while preoperative shock and longer bypass times decreased it [16].

A growing body of evidence indicates that social determinants of health (SDOHs) influence both the likelihood of achieving TO/TOO and survival after lung cancer surgery. Socioeconomic disadvantage reduced TO/TOO achievement and overall survival independent of stage and treatment, underscoring the need for equity-sensitive risk adjustment when benchmarking programs [20].

### 3.4. Prognostic Impact

Across cardiothoracic procedures, TO achievement consistently portends better outcomes (Table 3). In NSCLC, TO predicted improved overall survival independent of stage [5,6]. In esophagectomy, TO achievement appeared to be associated with significantly improved five-year overall, disease-free, and recurrence-free survival, with hazard ratios ~0.60–0.67 [7], and programs adopting robotic approaches achieved higher TO rates alongside long-term gains [19]. In lung transplantation, TO achievement was linked to superior patient and graft survival in single-center and national cohorts; failure to achieve TO carried substantially higher hazards of mortality and graft failure in UNOS analysis [8,9,15]. In adult heart transplantation, TO appeared to be associated with lower long-term mortality in both OPTN/UNOS studies; conditional on surviving for one year, TO conferred a large survival advantage (HR 0.55) [17,18]. In Norwood, TO tracked with lower costs and reduced long-term mortality [16]. These convergent findings position TO as a clinically meaningful surrogate for the overall quality of the operative episode and the system that supports it.

### 3.5. Health Economics and Benchmarking

Composite success correlates with resource efficiency. In lung transplantation, TO achievers incurred markedly lower inpatient charges than non-TO patients in institutional data [8]. In NSCLC, TO was associated with shorter stays and lower expenditures [6]. In Norwood, achieving TO correlated with significantly lower direct and total costs at the encounter level [16]. At the systems level, TO enables robust benchmarking. Population-based audits demonstrated wide program-level variation in TO after lung cancer resection and esophagectomy [5,10]. The UNOS lung transplant analysis showed adjusted center-level TO rates spanning from 27% to 72% [15], and adult heart transplant studies reported similarly broad program-level ranges, with TO outperforming one-year survival in discriminating program performance [17,18]. Notably, the UNOS lung transplant TO rate (~52%) contrasted with single-center estimates (~24%). This divergence likely reflects definition and ascertainment differences (registry-fixed 72 h ventilatory/graft endpoints) and case mix (pre-transplant support and bilateral procedures). We highlight this as a central example of why standardization is a prerequisite for fair benchmarking. These observations suggest that TO is a more sensitive, comprehensive, and actionable benchmark than survival alone.

## 4. Discussion

### 4.1. Principal Findings

The present review suggests that TO represents a pragmatic, clinically meaningful composite quality metric across cardiothoracic surgery. It integrates technical performance, perioperative management, and system factors into a single endpoint that is strongly associated with long-term outcomes and resource utilization. TO’s appeal lies in its clinical face validity, patient-centeredness, and benchmarking utility.

The most consistent signal across procedures is prognostic: patients who experience a “textbook” course have significantly enhanced long-term outcomes. This is evident in oncologic thoracic surgery, where TO predicts overall and disease-free survival [5,6,7], and in transplantation and congenital cardiac surgery, where TO maps to graft/patient survival and value [8,15,16,17,18]. These findings align with experiences from other specialties in which TO correlates with survival and lower recurrence or readmission [21,22,23]. Clinically, TO offers a tangible, shared goal for multidisciplinary teams and a communicable endpoint for patients.

### 4.2. Why TO Is Clinically Useful

Traditional surgical indicators, such as thirty-day mortality or single complication rates, are indispensable but limited as sole measures of quality. Mortality no longer discriminates in many high-volume centers; complication coding is variable and siloed. TO captures success across multiple dimensions, encompassing absence of serious complications, oncologic adequacy where relevant, timeliness of discharge, and early convalescence. It thus functions as an integrative, patient-centered metric and aligns with the broader move toward composite outcomes in surgery [4,24,25].

### 4.3. Sources of Heterogeneity and What They Mean

The principal barrier to wider adoption is variability in definitions across centers and registries. Thoracic definitions sometimes diverge on nodal thresholds or length-of-stay cut-offs; lung and heart transplant frameworks incorporate graft-specific milestones and 1-year functional outcomes [7,8,9,15,17,18]. The Norwood composite is operation-specific and consensus-driven [16]. Certain discrepancies, such as single-center lung transplant TO ~24% versus national UNOS ~52%, likely reflect differences in construct definition (e.g., fixed 72 h graft/ventilatory endpoints), ascertainment constrained by registry fields, and case mix [8,9,15]. Two imperatives are as follows: cross-cohort comparisons require explicit attention to construct validity and consistent risk adjustment; and harmonized, procedure-specific core TO sets, mapped to widely available STS and OPTN/UNOS fields, are essential for fair benchmarking [3,10,11,26,27,28,29].

### 4.4. Equity, Social Determinants, and Risk Adjustment

As TO moves toward benchmarking and potentially payment, risk adjustment must evolve beyond clinical covariates to incorporate social determinants of health (SDOH). Emerging data indicate that SDOH, including race/ethnicity, insurance status, and area-level deprivation, reduce the likelihood of TO/TOO and correlate with survival after lung cancer surgery. Equity-sensitive benchmarking will likely require explicit inclusion of SDOH covariates (e.g., race/ethnicity, payer, and an Area Deprivation Index) in risk-adjustment models, accompanied by transparent reporting of case mix and O:E rates. We caution that this adjustment must not normalize lower expectations for disadvantaged groups; instead, it should identify structural gaps and guide targeted interventions (navigation, language services, early rehab access) to raise TO attainment equitably. In fact, socioeconomic disadvantage demonstrably reduces TO/TOO achievement and survival in lung cancer surgery [20]. Without equity-sensitive models, TO could inadvertently penalize centers caring for disadvantaged populations. Incorporating SDOH into models, transparently reporting case mix–adjusted observed-to-expected (O:E) TO rates, and coupling TO with improvement supports (navigation programs, enhanced recovery access) will be essential to ensure fairness and to target disparities [20,26,27,28]. On the other hand, its implementation in the context of Quality Collaboratives, like the Michigan Society of Thoracic and Cardiovascular Surgeons Quality Collaborative (MSTCVS-QC) is critical to promote benchmarking and better outcomes [29,30,31].

Operationally, the determinants highlighted here identify specific, measurable targets: adequate nodal assessment in NSCLC; operative time efficiency and team ergonomics in MIE; donor–recipient optimization and ischemic time minimization in transplantation; and preoperative stabilization in Norwood and adult heart transplantation. Embedding TO into perioperative dashboards can focus teams on modifiable elements; coupling TO with process measures (e.g., time to extubation, early mobilization, standardized lymphadenectomy) may accelerate learning. Importantly, TO outperforms one-year survival in discriminating program performance in adult heart transplantation, supporting TO’s superiority in quality conversations [17,18].

### 4.5. Implementation Playbook (Kotter-Guided)

**Figure 3:**
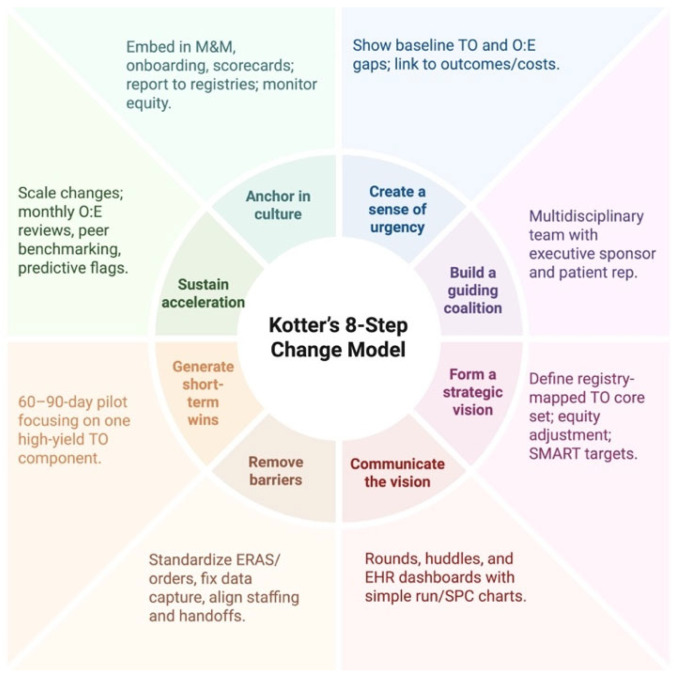
Implementing Textbook Outcome (TO) with Kotter’s eight steps. Created in BioRender, Magouliotis, D. (2025), https://BioRender.com/j2funze, accessed on 20 September 2025 [32].

To implement TO in everyday practice, programs can adapt Kotter’s eight steps [32] into a pragmatic roadmap (Figure 3): create urgency by sharing local baseline TO rates and center-to-center variation alongside concrete clinical vignettes to underscore the gap; build a guiding coalition that spans surgeons, anesthesia, perfusion, ICU, ward nursing, oncology/transplant medicine, case management, informatics, and finance; craft a clear vision that defines procedure-specific, registry-mapped TO sets with equity-sensitive risk adjustment and explicit links to patient-reported outcomes; communicate that vision relentlessly through grand rounds, unit huddles, dashboards embedded in the EHR, and patient-facing materials; remove barriers by standardizing order sets and ERAS pathways, fixing data capture and coding, aligning scheduling and staffing for lymph node adequacy or early extubation, and providing targeted training; generate short-term wins via 90-day pilots on a single service (e.g., lobectomy or heart transplant) with visible improvements in one or two TO components; consolidate gains by scaling to adjacent procedures, hard-wiring feedback loops (SPC charts, monthly O:E TO reviews), and aligning incentives in service line scorecards; and finally anchor the change in culture by making TO part of M&M review, credentialing and onboarding, annual program goals, and contributions to national audits, so that “textbook” care becomes the expected norm rather than the exception.

Below is the Implementation Playbook (Kotter-guided):

- Create urgency: share local baseline TO and O:E gaps; pair with clinical vignettes;
- Build a coalition: surgeon, anesthesia, ICU, ward nursing, oncology/transplant, case management, informatics, finance, patient rep;
- Form a vision: define a registry-mapped TO core set per procedure; prespecify equity adjustment; set SMART targets;
- Communicate: rounds/huddles; simple run/SPC charts on the EHR dashboard;
- Remove barriers: standardize ERAS/order sets; fix data capture; align staffing/handoffs;
- Generate short-term wins: a 60–90-day pilot focused on one high-yield TO component;
- Sustain acceleration: monthly O:E reviews; peer benchmarking; predictive TO-risk flags;
- Anchor in culture: embed in M&M, onboarding, and scorecards; report to registries; monitor equity.

### 4.6. Limitations of the Evidence

The synthesized literature is observational and subject to confounding and selective reporting, with multiple studies judged as showing a moderate to serious risk of bias according to ROBINS-I, particularly for selection of the reported result and outcome measurement. TO definitions were heterogeneous, and some components (e.g., lymph node adequacy, length-of-stay cut-offs) vary across datasets and health systems. These constraints limit causal inference and suggest that practice recommendations should be interpreted as conditional pending prospective validation and consensus-based standardization.

### 4.7. Future Directions and Deliverables

Future work should prioritize three aspects. First, there is standardization: studies should convene STS/ESTS/ISHLT and congenital consortia to define core TO sets per procedure, with narrowly tailored additions and publish mapping to registry fields to ensure reproducibility [11,26,31]. We propose a Delphi consensus led jointly by STS, ESTS, and ISHLT (and congenital societies) to define procedure-specific core TO sets with explicit registry mapping and SDOH adjustment guidance. A parallel prospective registry validation should evaluate the discrimination, calibration, and value (cost) of each TO set. Second, patient-centeredness must be valued through the integration of patient-reported outcomes as adjuncts or next-generation composites and the pairing of TO with continuous morbidity metrics such as the Comprehensive Complication Index to capture gradations of harm [19]. Third, for evaluation and implementation, TO should be prospectively validated in registries and trials, and TO-based quality improvement bundles and study value should be tested by linking TO to cost and payment models. Advanced analytics, such as real-time dashboards, prediction models for “TO risk”, and digital pathways, can facilitate preemptive mitigation. Equity must be embedded from the outset, with SDOH-aware risk adjustment and transparent reporting to avoid unintended consequences [27,28].

## 5. Conclusions

TO appears to be a pragmatic, patient-centered composite that correlates with long-term outcomes and resource use across cardiothoracic procedures. It captures the essence of high-quality care (safe, thorough operations; uneventful recovery; timely discharge) while correlating strongly with survival, graft durability, and value. Its utility in benchmarking is evident: TO exposes between-center variability that is not apparent when relying on mortality alone, especially in domains like adult heart transplantation where one-year survival is tightly clustered. The field’s next step is standardization: it is necessary to develop procedure-specific core TO sets aligned with STS and OPTN/UNOS fields, integrate equity-sensitive risk adjustment, and validate TO prospectively. Given observational evidence and heterogeneous definitions, we propose focusing next on consensus-based standardization (registry-mapped core sets), equity-sensitive risk adjustment, and prospective validation. Embedding TO in perioperative dashboards and QI collaboratives could help teams target modifiable drivers and reduce failure modes while avoiding unintended disparities. With harmonized definitions, patient-reported outcomes, and equity at its core, TO can serve as a unifying, patient-centered benchmark that guides clinical practice, quality improvement, and policy across cardiothoracic surgery.

## Figures and Tables

**Table 2:** Incidence of textbook outcome across cardiothoracic procedures.

Procedure	Study/Cohort	N	TO rate	Leading Drivers of Failure
NSCLC resection	Dutch Lung Cancer Audit	5513	26.4%	Inadequate nodal dissection [5]
NSCLC resection	Korean single-center study	418	66.3%	Prolonged air leak; prolonged LOS [6]
Esophagectomy (MIE)	China	528	53.2%	Postoperative complications [7]
Esophagectomy (MIE)	Two-center study (EU/Asia)	945	46.6%	Complications; inadequate lymphadenectomy [19]
Esophagectomy (MIE)	High-volume center	2210	40.8%	Prolonged operative time; complications [14]
Lung transplantation	Academic single-center study	401	24.2%	Delayed extubation [8]
Lung transplantation (US registry)	UNOS, 2016–2019	8959	52.1%	Intubation at 72 h; LOS >30 d; ventilation ≥5 d; PGD3 at 72 h [15]
Norwood operation (congenital)	Single-center study, 2005–2021	196	30%	Prolonged ventilation (49%); reintubation (46%); prolonged LOS (30%) [16]
Heart transplantation (adult)	OPTN/UNOS, 2005–2017	24,620	45.4%	Treated rejection during index stay; one-year rejection [17]
Heart transplantation (adult)	OPTN/UNOS, 2011–2022	26,885	37%	Early ECMO; prolonged LOS; PGD; 1 y EF ≤50%; readmission for rejection/infection [18]

Abbreviations: MIE, minimally invasive esophagectomy; LOS, length of stay; UNOS, United Network for Organ Sharing; PGD3, primary graft dysfunction grade 3; ECMO, extracorporeal membrane oxygenation; EF, ejection fraction; EU, European Union.

**Table 3:** Predictors and prognostic impact of textbook outcome in cardiothoracic surgery.

Predictor/Effect	Setting	Adjusted Association
Older age; ASA ≥3; smoking; blood loss	Esophagectomy	Lower TO; worse OS/DFS [6,7]
Operative time >~298 min (inverse-U)	Esophagectomy (MIE)	Reduced TO beyond peak time [14]
Inadequate lymph node dissection	NSCLC resection	Strong determinant of non-TO [5]
Male sex; low DLCO	NSCLC lobectomy	Higher risk of non-TO [6]
Robotic vs. thoracoscopic approach	Esophagectomy	Higher TO with robotic [19]
Planned VA-ECMO vs. off-pump	Lung transplantation	Higher TO with planned ECMO [9]
Pretransplant ventilation/ECMO	Lung transplantation (UNOS)	Lower odds of TO; adverse case mix effects [15]
DCD donor; ischemic time (per hour); obesity; non-White race	Lung transplantation (UNOS)	Lower odds of TO; equity and donor factors [15]
Single- vs. bilateral transplant	Lung transplantation (UNOS)	Higher odds of TO with single lung [15]
TO achievement → patient/graft survival	Lung transplantation	Lower hazards of death and graft failure with TO [8,9,15]
Greater weight; absence of shock; shorter CPB	Norwood	Higher odds of TO [16]
TO achievement → lower costs	Norwood	Lower direct/total costs with TO [16]
No preop MCS/dialysis; not hospitalized	Heart transplant	Higher odds of TO [17,18]
TO achievement → long-term survival	Heart transplant	Substantially lower mortality hazards [17,18]

Abbreviations: OS, overall survival; DFS, disease-free survival; MIE, minimally invasive esophagectomy; DLCO, diffusing capacity of the lung for carbon monoxide; VA-ECMO, venoarterial extracorporeal membrane oxygenation; UNOS, United Network for Organ Sharing; DCD, donation after circulatory death; CPB, cardiopulmonary bypass; MCS, mechanical circulatory support.

## Data Availability

The data that support the findings of this study are available from the corresponding author upon reasonable request.

## Supplementary Materials

The following supporting information can be downloaded at: https://www.mdpi.com/article/10.3390/jcm14217660/s1, File S1: PRISMA Checklist; File S2: Full database search strings and language filters.
