# Supplementary material for: Beyond Mortality: Textbook Outcome as a Novel Quality Metric in Cardiothoracic Surgical Care
Source: J Clin Med. 2025 Oct 28;14(21):7660. doi: 10.3390/jcm14217660 (PMC12609948; doi:10.3390/jcm14217660)
Supplement: Supplementary file 1 [file jcm-14-07660-s001.zip › jcm-3914623-supplementary/jcm-3914623-supplementary File S2.pdf]

Supplementary Materials

Section S1. Full database search strategies (final run: 1 Jan 2010–19 Sep 2025; English only)

Section S1.1. MEDLINE (via PubMed)

```
"textbook outcome"[tiab] OR "textbook outcomes"[tiab] OR "composite outcome"[tiab] OR "composite outcomes"[tiab] OR "textbook oncologic outcome"[tiab] OR "TOO"[tiab]
AND
thoracic[tiab] OR "thoracic surgery"[tiab] OR "lung cancer"[tiab] OR NSCLC[tiab] OR esophagectomy[tiab] OR esophageal[tiab] OR "lung transplant*"[tiab] OR "heart transplant*"[tiab] OR "congenital heart"[tiab] OR Norwood[tiab] OR cardiothoracic[tiab] OR "cardiac surgery"[tiab]
Filters: English; publication date from 2010/01/01 to 2025/09/19.
```

Rationale: keyword strategy favoring high recall across heterogeneous TO/TOO terminology and cardiothoracic procedures.

Section S1.2. Scopus (Title/Abstract/Keywords)

```
TITLE-ABS-KEY ("textbook outcome" OR "textbook outcomes" OR "composite outcome" OR "textbook oncologic outcome" OR TOO)
AND TITLE-ABS-KEY (thoracic OR "thoracic surgery" OR "lung cancer" OR NSCLC OR esophagectomy OR esophageal OR "lung transplant*" OR "heart transplant*" OR "congenital heart" OR Norwood OR cardiothoracic OR "cardiac surgery")
AND (PUBYEAR > 2009 AND PUBYEAR < 2026)
AND (LIMIT-TO (LANGUAGE, "English")).
```

Section S1.3. Cochrane Central (CENTRAL)

```
("textbook outcome" OR "composite outcome" OR "textbook oncologic outcome" OR TOO):ti,ab,kw
AND (thoracic OR "thoracic surgery" OR "lung cancer" OR NSCLC OR esophagectomy OR esophageal OR "lung transplant*" OR "heart transplant*" OR "congenital heart" OR Norwood OR cardiothoracic OR "cardiac surgery"):ti,ab,kw
Limits: 2010–2025; English.
```

Section S1.4. Grey literature and supplementary sources

Abstract books from ESTS, STS, and ISHLT within the date range; forward/backward citation chaining for all included studies (Google Scholar); ClinicalTrials.gov and WHO ICTRP keyword searches for "textbook outcome" and "composite outcome" within surgery (2010–2025).

Section S1.5. De-duplication and screening workflow

Records exported (RIS/CSV) and de-duplicated in EndNote/Zotero using Title + First Author + Year matching, then manually verified. Title/abstract screening in duplicate

(Rayyan, blinded), followed by full-text screening in duplicate. Disagreements resolved by consensus with senior arbitration. PRISMA 2020 reporting followed [12].

Section S1.6. Inclusion/exclusion anchors

Include human studies in cardiothoracic surgery that define and report Textbook Outcome (TO) or Textbook Oncologic Outcome (TOO) and provide incidence, determinants, benchmarking, or outcome associations (survival, graft, cost). Eligible designs: cohorts, registries, propensity-matched analyses, randomized trials.

Exclude non-cardiothoracic procedures, n < 10, editorials or narrative reviews without primary data, non-English reports, studies without a TO/TOO construct, and overlapping cohorts (retain the most complete/recent).

Section S2. PRISMA flow and checklist materials

Section S2.1. PRISMA flow numbers (to be completed)

• Records identified via databases (MEDLINE, Scopus, CENTRAL): n = ____.

• Additional records from grey literature/citation chaining: n = ____.

• After de-duplication: n = ____.

• Title/abstract screened: n = ____; excluded: n = ____.

• Full texts assessed: n = ____; excluded with reasons: n = ____.

• Studies included in qualitative synthesis: n = 10.

• Studies included in quantitative synthesis (if any): n = ____.

Section S2.2. PRISMA 2020 checklist

The completed PRISMA 2020 checklist is provided separately and references the exact manuscript locations for each item (Methods/Supplementary sections as applicable) [12].

Section S3. Data extraction form (fields captured)

First author; year; country; design; setting; sample size; procedure (NSCLC resection, esophagectomy, lung transplantation, Norwood, adult heart transplantation); surgical approach (open, VATS, robotic; single vs bilateral transplant); TO definition (all components and thresholds, including any 1-year elements for transplant frameworks); TO incidence and top failure modes; patient/donor covariates (age, sex, ASA, DLCO, preoperative ventilation/ECMO, ischemic time, DCD donor, comorbidity); system/process measures (operative time, lymph-node adequacy, ERAS use); effect estimates (OR/HR, covariates); resource use (LOS, readmission, costs/charges); center-level benchmarking (O:E TO, volume, between-center variation); follow-up; funding; conflicts.

Section S4. Operationalization of TO by procedure and registry mapping

NSCLC resection. Core TO: R0 resection; adequate lymph-node dissection; no in-hospital/30-day mortality; no major complications or reintervention; no ICU readmission; no prolonged LOS; no 30-day readmission. Mapping: institutional EMR + STS General Thoracic fields.

Esophagectomy (MIE/open). Core TO: R0; ≥15 nodes; no major complications; no ICU readmission; LOS ≤21 days; no 30-day readmission. Mapping: institutional EMR and upper-GI audit; operative time from OR record.

Lung transplantation (single-center). Core elements: early extubation (≤48 h); no PGD3 at 72 h; no ECMO/dialysis; no early rejection; no reintubation/tracheostomy ≤7 days; no major in-hospital complications [8,9]. Mapping: transplant EMR; ISHLT PGD grading; ICU ventilatory/perfusion records.

Lung transplantation (US registry, UNOS). Freedom from: intubation at 72 h; ECMO at 72 h; mechanical ventilation ≥5 days; PGD3 at 72 h; inpatient dialysis; airway dehiscence; 90-day mortality; index LOS >30 days; 30-day readmission; pre-discharge acute rejection [15]. Mapping: OPTN/UNOS standardized elements.

Norwood (congenital). Survival without ECMO, cardiac arrest, reintubation, or reintervention; no 30-day readmission; invasive ventilation <10 days; index LOS <66 days [16]. Mapping: STS Congenital database + EMR.
Adult heart transplantation (OPTN/UNOS). Ten-item construct: index LOS ≤30 days; no stroke/dialysis/treated rejection; 1-year: EF >50%, Karnofsky 80–100%, and no treated rejection/graft failure/chronic dialysis/re-transplant/death [17]. Six-domain construct: no ECMO ≤72 h; LOS <21 days; no postoperative stroke/pacemaker/dialysis; no PGD; no 1-year readmission for rejection/infection/re-transplant; EF >50% at 1 year [18].

Section S5. Risk of bias assessment (ROBINS-I decision rules) [13]

Confounding. Rate serious if no adjustment for key covariates (e.g., age, comorbidity, stage/approach; in transplant: preoperative ventilation/ECMO, ischemic time, DCD donor); moderate if multivariable/propensity adjustment is plausible; low rarely applicable in observational surgery.

Selection of participants. Moderate for population registries with standard inclusion; serious for single-center highly selected cohorts or unclear post-baseline exclusions.

Classification of interventions. Low when procedural categories are objective and prospectively recorded (e.g., robotic vs thoracoscopic; single vs bilateral transplant); moderate if retrospective/mixed.

Deviations from intended interventions. Moderate in observational surgical series; ERAS/protocolization may mitigate.

Missing data. Low if completeness >90% for primary TO components and mortality; moderate/serious if key TO fields (e.g., PGD3, readmission) are frequently or differentially missing.

Measurement of outcomes. Low for objective/registry-coded outcomes (mortality, LOS, extubation time, dialysis, EF); moderate where subjective complication grading dominates without adjudication.

Selection of the reported result. Moderate if protocol not available; low if statistical plan or registry-mapped outcome set is pre-specified.

Two reviewers assess independently; discrepancies resolved by consensus. Domain-level summaries are presented in the main text.

Section S6. Synthesis and analysis plan

Primary approach. Narrative synthesis across procedures: definitions; incidence and failure modes; determinants (patient, intraoperative, system); prognostic and economic impact; benchmarking.

Quantitative synthesis (conditional). Meta-analysis only for homogeneous subsets sharing a procedure and TO definition. Random-effects models (REML or DerSimonian–Laird with Hartung–Knapp adjustment); pooled OR/HR with 95% CIs. Convert medians/IQRs to means/SDs only when necessary and appropriate. Heterogeneity assessed with I^2, τ^2, and Cochran's Q; small-study effects via funnel plot and Egger's regression when ≥10 studies. Leave-one-out sensitivity analyses where feasible.

Pre-specified subgroups. Procedure (NSCLC, esophagectomy, lung transplant, Norwood, heart transplant); approach (open, VATS, robotic; single vs bilateral); geography; registry versus single-center design.

Equity lens. When available, summarize SDOH associations with TO/TOO and survival; highlight the need for equity-sensitive risk adjustment in benchmarking [19,31].

Section S7. Data items for reproducibility (minimum dataset available on request)

For each included study: full citation; design/timeframe; inclusion criteria; exact TO definition (all components/thresholds); sample size; TO incidence; top three failure modes; adjusted predictors (variables and effect sizes); outcome associations (OS/DFS/graft survival/costs); center-level metrics (O:E TO, volume, between-center variation); and risk-of-bias summary.

Section S8. Abbreviations used in Supplementary Materials

TO, textbook outcome; TOO, textbook oncologic outcome; NSCLC, non-small cell lung cancer; MIE, minimally invasive esophagectomy; LOS, length of stay; STS, Society of Thoracic Surgeons; OPTN/UNOS, Organ Procurement and Transplantation Network/United Network for Organ Sharing; PGD3, primary graft dysfunction grade 3; DCD, donation after circulatory death; ECMO, extracorporeal membrane oxygenation; O:E, observed-to-expected; SPC, statistical process control; SDOH, social determinants of health.
